# Use of a new vertical traction device for early traction-assisted staged closure of congenital abdominal wall defects: a prospective series of 16 patients

**DOI:** 10.1007/s00383-024-05745-6

**Published:** 2024-07-03

**Authors:** Anna-Maria Ziegler, Daniel Svoboda, Britta Lüken-Darius, Andreas Heydweiller, Fritz Kahl, Sophie Christine Falk, Udo Rolle, Till-Martin Theilen

**Affiliations:** 1 Department for Pediatric Surgery, University Medical Center, Bonn, Germany; 2 Department for Pediatric Surgery, University Medical Center, Mannheim, Germany; 3 Department of Pediatric Surgery and Pediatric Urology, University Hospital Frankfurt, Theodor-Stern-Kai 7, 60590 Frankfurt/M., Germany; 4 Department for Pediatric Surgery, University Medical Center, Göttingen, Germany

**Keywords:** Giant omphalocele, Gastroschisis, Primary closure, Viscero-abdominal disproportion, Fascial traction

## Abstract

**Purpose:**

Abdominal wall closure in patients with giant omphalocele (GOC) and complicated gastroschisis (GS) remains to be a surgical challenge. To facilitate an early complete abdominal wall closure, we investigated the combination of a staged closure technique with continuous traction to the abdominal wall using a newly designed vertical traction device for newborns.

**Methods:**

Four tertiary pediatric surgery departments participated in the study between 04/2022 and 11/2023. In case primary organ reduction and abdominal wall closure were not amenable, patients underwent a traction-assisted abdominal wall closure applying fasciotens^®^Pediatric. Outcome parameters were time to closure, surgical complications, infections, and hernia formation.

**Results:**

Ten patients with GOC and 6 patients with GS were included. Complete fascial closure was achieved after a median time of 7 days (range 4–22) in GOC and 5 days (range 4–11) in GS. There were two cases of tear-outs of traction sutures and one skin suture line dehiscence after fascial closure. No surgical site infection or signs of abdominal compartment syndrome were seen. No ventral or umbilical hernia occurred after a median follow-up of 12 months (range 4–22).

**Conclusion:**

Traction-assisted staged closure using fasciotens^®^Pediatric enabled an early tension-less fascial closure in GOC and GS in the newborn period.

## Introduction

Omphalocele (OC) and gastroschisis (GS) are the most common congenital abdominal wall defects. The prevalence is between 1.0 and 3.8 per 10,000 live births for OC and 2.6–5.1 per 10,000 live births for GS in the United States and Europe [1–3]. Treatment of OC and GS consists of a complete reduction of the externalized organs, optimally followed by a complete closure of the abdominal wall including the fascia and skin. However, in about 50–70% of cases in OC and in up to 30% of cases in GS, a primary single-stage reconstructive closure of the abdomen including the fascia is not achieved. These cases require a staged or delayed closure [4, 5].

Abdominal wall closure in OC and GS depends on the size of the abdominal wall defect, the volume of the externalized viscera and the amount of space within the abdominal cavity. Cases of OC with organ protrusion limited to bowel (hernia to the cord) and most cases of uncomplicated GS can usually be closed primarily in a single-stage procedure. However, giant omphaloceles (GOC), commonly defined by a defect size greater than 5cm and more than 50% of the liver volume in the amniotic sac, and GS with a great bulk of edematous stiff bowel loops outside of the abdomen are typical entities for a multi-step closure approach [6, 7]. These cases have a great discrepancy between a large organ volume outside of the abdomen and a small intra-abdominal domain (viscero-abdominal disproportion) [8]. In addition, the abdominal wall has undergone retraction and hypotrophy due to the lack of its distension by growing intraabdominal organs during fetal development. In these cases, organ reduction and (surgical) closure of the abdomen can become extraordinarily challenging.

Multiple different operative and non-operative approaches have been described to overcome the surgical difficulties in complex cases [4, 9–12]. The choice of treatment usually depends on the medical status of the patient, the availability of operative and intensive medical care and the preference of the surgeon. One of the most prominently applied operations is the staged closure technique first described by Schuster [4]. Multiple different variations of this technique have been described [13–17]. In this multi-step procedure, the eviscerated organs are placed in a tailored prosthetic silo sutured to the abdominal wall or in a preformed (spring-loaded) silo bag anchored underneath the fascia. Organs gradually decent into the abdomen by gravity force and by progressive plication of the silo [14–17].

In some cases, fascial closure still fails due to non-resolvable viscero-abdominal disproportion, or complications such as abdominal compartment syndrome with potential failure of respiratory, cardiac and kidney function [18]. In these individual cases, a closing layer above the abdominal wall defect can be achieved by inducing epithelization (“paint and wait”) of the amniotic sac in GOC, by skin and umbilical cord flaps or prosthetic and biological mesh implantation in both, GS and GOC [19–23]. These patients, however, may suffer from (severe) scaring around the abdominal wall defect and from the appearance of (large) ventral or umbilical hernias [24–26]. Further surgery for definitive abdominal wall closure or repair of residual umbilical hernias is typically done later in life for these patients [27–29].

Among all treatment options, achieving complete closure of the abdominal wall usually offers the best result in regard to functionality (organ protection, body stability) and aesthetics [9, 30]. To increase the chances for an early complete fascial closure, fascial traction has been added to the staged closing procedure either by simple suspension of the silo bag to the top of the incubator or by active external traction [31–35]. In reference to these traction-assisted closure techniques, we investigated the application of a newly developed, CE certified traction device, fasciotens^®^Pediatric, in newborns with GOC and GS in a multicenter prospective feasibility study. The aim of this study was to investigate the safety and outcome in the application of a controlled and quantifiable traction to the abdominal wall.

## Methods

A multicenter prospective feasibility study was performed, collecting clinical data on the application of standardized vertical traction to the abdominal wall using fasciotens^®^Pediatric (fasciotens GmbH, Essen, Germany) in newborns with GOC and GS. The study included 16 patients treated at 4 different tertiary university hospitals in Germany between April 2022 and November 2023 (contributing institutions: University Hospital Bonn (6 patients), University Medical Centre Mannheim (4 patients), University Medical Centre Göttingen and University Hospital Frankfurt (3 patients each)). The following variables were collected during this study: age, sex, body weight, diagnosis, defect type, force and duration of traction, surgical or mechanical complications during and after the traction treatment (surgical site occurrence (SSO) and surgical site infections (SSI)), duration of mechanical ventilation, duration of intensive care admission, and duration of hospital stay.

### Inclusion and exclusion criteria

Patients with OC and GS were included in case of a great viscero-abdominal disproportion not allowing the positioning of the externalized organs into the abdominal cavity at the primary intervention. Patients with abdominal wall aplasia, coagulation disorders, and cardiorespiratory instability including pulmonary hypertension, pulmonary hypoplasia, and severe cardiac defects were excluded.

### Perinatal and surgical management

**Fig. 1:**
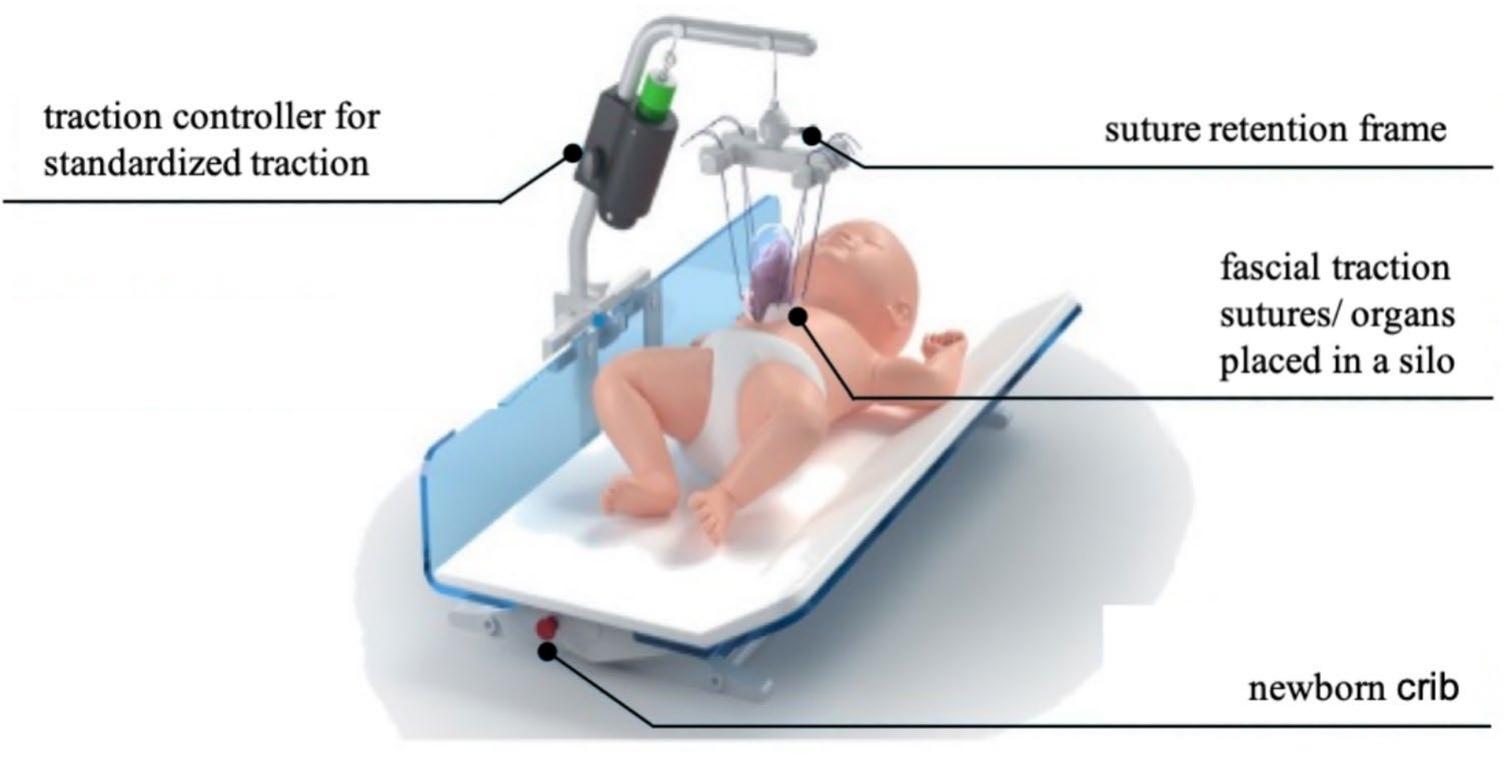
Schematic illustration of the fasciotens®Pediatric device (figure modified with approval by Fasciotens GmbH, Essen, Germany)

All patients were born by planed Caesarian section and initially stabilized on the neonatal intensive care unit (NICU) after birth. In cases, in which primary repositioning of the exteriorized organs and closure of abdominal wall was not feasible, a modified staged silo procedure with vertical traction of the abdominal fascia was chosen. Standardized continuous traction was applied to the fascia using the fasciotens^®^Pediatric traction device (Fasciotens GmbH, Germany; Fig. 1). The mode of application of the traction device to the fascia varied between the participating institutions. The device was either applied under general anesthesia or under short analgesic sedation without mechanical ventilation at the bedside. In 11 patients, a synthetic mesh made of Vicryl™, Gore-Tex®, or silicone was attached to the fascia with non- absorbable sutures. In 5 GOC patients the amniotic sac was not resected and a Gore-Tex® mesh or single traction sutures were fixed transcutaneously into the fascia at the rim of the abdominal wall defect. Depending on the size of the defect, 4 to 8 traction sutures (Vicryl^TM^Plus, USP 2, Ethicon®, USA) were used. During the traction treatment the abdominal organs were temporarily covered with a tailored or a preformed spring-loaded silo or by the amniotic sac. The newborn was placed into a specially designed newborn crib (fasciotens^®^Cradle, Fasciotens GmbH, Germany) and the traction sutures were connected to the suture retention frame of the traction device. The traction controller was set between 500 and 1000 g (< 50% of the patient’s weight) of traction force. The traction force was limited to less than 50% of the patient’s weight in accordance with a previous report [31]. The traction progress was evaluated at the bedside on a daily basis. In each hospital, the same surgeon performed and supervised the device application during the treatment. Thirteen out of 16 patients were under general anesthesia (GA) with muscle paralysis and mechanical ventilation during the traction procedure. Three patients did receive sedation, muscle paralysis or ventilation during the traction procedure. As soon as organs had descended into the abdominal domain and fascial closure appeared possible by the surgeon’s assessment at the bedside, abdominal wall traction was terminated and the abdominal wall including fascia and skin was closed in the operating room. After the operation, patients were weaned from GA and the ventilator. Abdominal pressure was monitored by secondary clinical parameters including ventilation pressure, cardiac function, and urine output.

### Data analysis

Data collection was conducted by every hospital independently and transmitted pseudonymously to the study center. The classification for surgery-related complications were divided into two categories, as is commonly used in abdominal wall surgery in adults and proposed by Haskins et al. [36]. Infections related to the surgical procedure were categorized as Surgical Site Infections (SSI). Surgical Site Occurrence (SSO) was used for a wider spectrum of surgery related complications (e.g., wound healing disorders, fascial disruption, skin/soft tissue necrosis, e.g.). Data were expressed as mean and standard deviation or median and range. Differences between groups were calculated using the Student’s t-test with a level of significance of *p* < 0.05. Central data collection and analysis was done using Microsoft Excel.

### Ethical approval

This study was approved by the institutional review board of the University Hospital Frankfurt (No. 20–659).

## Results

Ten patients with COG and 6 patients with simple GS (without of bowel atresia) were treated with fascial traction using fasciotens^®^Pediatric for abdominal wall closure. Six patients were born preterm. The youngest patient age was 34 + 4 weeks of gestation and the lowest patient weight was 1500g (Table 1 and 2).

**Table 1:** Overview of patients’ characteristics, treatment modalities and outcome of patients with giant omphalocele treated with fasciotens^®^Pediatric

Patient characteristics				Treatment modalities					Outcome			
Case No.	Sex	GA at birth (weeks + days)	Birth weight (gram)	Traction sutures applied to	Temporary enclosure of organs by	Fascial traction^#^ (days)	Traction force (gram)	Ratio of traction force to body weight (%)	SSI/ SSO during traction	Ventilation (days)	NICU admission (days)	Length of hospital stay (days)
1	m	37 + 1	2070	Vicryl mesh	Spring loaded silo	8	500	24.0	No	32	137	137
2	m	38 + 4	3250	Vicryl mesh	Spring loaded silo	13	500	16.0	No	16	37	37
3	m	39 + 0	3500	Vicryl mesh	Spring loaded silo	5	1000	29.0	No	8	11	31
4	m	36 + 4	2900	Gore-Tex^®^ patch	Amniotic sac*	22	1000	34.0	Yes**	46	73	73
5	f	34 + 5	2700	Gore-Tex^®^ patch	Amniotic sac*	5	750	28.0	No	18	28	28
6	m	35 + 6	3000	Gore-Tex^®^ patch	Amniotic sac*	6	1000	33.0	No	24	34	34
7	m	37 + 1	3740	Tailored silicone silo	Tailored silicone silo	4	750	20.0	No	7	10	17
8	f	39 + 0	3510	Fascia	Amniotic sac*	20	1000	29.0	No	1****	27	31
9	m	38 + 0	3120	Fascia	Amniotic sac*	9	1000	32.0	Yes***	0*****	16	23
10	m	39 + 1	3300	Tailored silicone silo	Tailored silicone silo	5	1000	30,0	No	7	15	31

*F*   female, *m*   male, *GA*   gestational age, *SSI*   surgical site infection, *SSO*   surgical site occurrence, *NICU*   neonatal intensive care unit,

^#^Time to abdominal wall closure, *not resected initially, **Gore-Tex® patch tore out of fascia, ***traction sutures tore out of fascia, **** anesthesia with ventilation for one day at closure operation + no sedation, muscle paralysis and ventilation during traction, *****no sedation, muscle paralysis or ventilation during the traction procedure

**Table 2:** Overview of patients’ characteristics, treatment modalities and outcome of patients with gastroschisis treated with fasciotens®Pediatric

Patient characteristics				Treatment modalities					Outcome			
Case No.	Sex	GA at birth (weeks + days)	Birth weight (gram)	Traction sutures applied to	Temporary enclosure of organs by	Fascial traction^#^ (days)	Traction force (gram)	Ratio of traction force to body weight (%)	SSI/ SSO during traction	Ventilation (days)	NICU admission (days)	Length of hospital stay (days)
1	f	37 + 1	2200	Vicryl™ mesh	Spring loaded silo	5	1000	45.0	No	8	31	31
2	f	38 + 2	3500	Vicryl™mesh	Spring loaded silo	5	1000	29.0	No	5	30	30
3	f	36 + 6	1500	Vicryl™ mesh	Spring loaded silo	4	500	33.0	No	5	26	26
4	m	35 + 2	3100	Vicryl™ mesh	Spring loaded silo	7	1000	32.0	No	27	37	37
5	f	35 + 2	1870	Fascia	Tailored silicone silo	2	750	38.0	Yes*	12	16	62
6	m	35 + 6	2850	Fascia	Foil wound dressing	11	750	26.0	No	2**	16	32

*f*   female, *m*   male, *GA*   gestational age, *SSI*   surgical site infection, *SSO*   surgical site occurrence, *NICU*   neonatal intensive care unit

^#^time to abdominal wall closure, *skin dehiscence 2 days after abdominal wall closure, **anesthesia with ventilation for one day each at primary and closure operation + no sedation, muscle paralysis and ventilation during traction

**Fig. 2:**
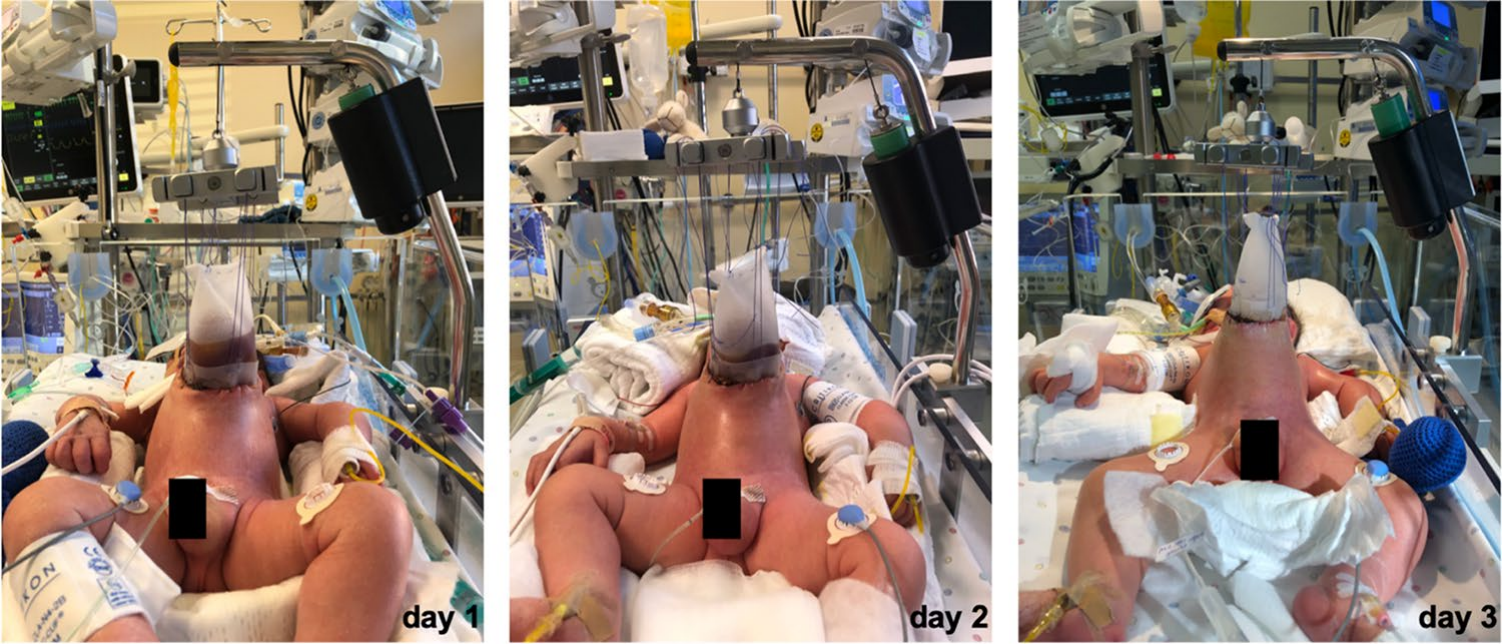
Application of fasciotens^®^Pediatric for traction-assisted staged closure in a giant omphalocele. Gradual descent of the liver and increasing distension of the abdominal wall shown from day 1 to day 3 of continuous traction with 1000g (30% of body weight) in a male newborn

In 4 cases, traction sutures were directly applied to the fascia. In all other cases, traction sutures were applied to alloplastic material made of Vicryl™, Gore-Tex® or silicone which had been sutured to the fascia. Figure 2 illustrates the application of a tailored silo with traction sutures in a patient with GOC (patient No. 10 of Table 1). The median traction force was 1000g (range 500–1000g) in both groups of GOC and GS. The median duration of fascial traction until fascial closure was 7 days (range 4–22 days) for GOC and 5 days (range 4–11 days) for GS. Correspondingly, patients with GS had less days of mechanical ventilation compared to patients with GOC. Median duration of NICU and hospital admission were similar between both entities (Table 3).

**Table 3:** Overview of patient and outcome data in patients treated with fasciotens®Pediatric for giant omphalocele and gastroschisis

	Giant omphalocele	Gastroschisis
Number of cases	10	6
Median age at birth* (range)	37 + 5 (34 + 5 to 39 + 1)	35 + 6 (32 + 2 to 38 + 2)
Median birth weight in gram (range)	3185 (2070–3740)	2525 (1500–3500)
Median traction force in gram (range)	1000 (500–1000)	1000 (500–1000)
Median ratio of traction force to body weight in % (range)	29 (16–35)	33 (26–45)
Median days of traction** (range)	7 (4–22)	5 (2–11)
Median days of ventilation (range)	12 (0–46)	7 (2–27)
Median days of NICU admission (range)	28 (10–137)	28 (16–37)
Median days of hospital stay (range)	31 (17–137)	32 (26–62)

*NICU* neonatal intensive care unit

*in weeks + days, **same as: median time to abdominal wall closure

In 5 out of 10 GOC cases, the amniotic sac was left in place during traction and was resected at the time of abdominal wall closure. In the remaining five cases the amniotic sac was resected and abdominal organs were enclosed by preformed spring-loaded or tailored silicone silos. The time to abdominal wall closure among cases without initial amniotic sac resection and with sac resection was statistically not different (9 days (range 5–22 days) vs. 5 days (range 4–13 days); *p* = 0.22).

In two GOC cases, a SSO was documented during the traction treatment. In one case the Gore-Tex® patch tore away from the abdominal wall and had to be re-attached. In one other case, traction sutures which were directly pierced to the rim of the defect migrated through the tissue during traction. In both cases, the amniotic sac had not been resected at the time of the initial operation. There were no SSO in cases in which the amniotic sac of the GOC had been resected primarily (Table 1).

Another SSO occurred after termination of the traction treatment in a patient with GS (Table 2). Two days after fascial and skin closure, there was a skin suture line dehiscence which had to be revised twice in the operating room. During the traction treatment of this patient, traction was only applied to the fascia excluding the skin. By the time of abdominal wall closure the skin was brought together under tension leading to the wound dehiscence. There was no case of SSI during or after the traction procedure and no compartment syndrome was seen.

The median follow-up time was 12 months (range 4–22 months). During this time, no hernia formation was found in any of the cases. None of the patients died during the treatment or in the course of the follow-up.

## Discussion

In this study, we present the first experience in traction-assisted abdominal wall closure in newborns with GOC and GS using the fasciotens®Pediatric traction device. The traction device was applied in 10 cases of GOC and 6 cases of GS. All patients underwent a staged closure of the defect in combination with continuous traction to the abdominal wall. Complete abdominal wall closure was achieved in all patients after a median time of 7 days (range 4–22 days) in GOC and of 5 days (range 4–11 days) in GS.

Overcoming the viscero-abdominal disproportion is one of the main therapy challenges in patients with congenital abdominal wall defects. Historically, the introduction of the staged closure technique with multiple variations of prosthetic silos between the late 1960s to the mid 1990s has added an innovative treatment option for patients with large defects [13–15, 17]. The silos, either handsewn or preformed, serve as a temporary extension of the abdominal cavity enclosing the displaced organs and protecting the newborn from heat and fluid loss. The silos are usually suspended to the top of the child’s incubator to keep it upright and folded daily to enhance the gravitational descent of the viscera into the abdominal cavity. The suspension and folding of the silo bag as well as the organ descent into the abdomen result in a progressive distension of the abdominal wall ideally enabling its closure. In some cases, the silo is followed by an interposition of an alloplastic patch at the level of the fascia with stepwise size reduction to further approximate the fascia lines.

### Traction-assisted staged closure

To enhance the abdominal distension, some surgeons have added external traction to the abdominal wall documenting that the abdominal wall in newborns is responsive to stretching procedures [31–35, 37]. The most prominent study by Patkovsky and colleagues included a series of 8 patients with OC and 21 patients with GS treated with external traction. Traction was applied via a pulley system and a full-thickness abdominal wall closure was achieved in less than 6 days in all patients [31]. In another publication, Svetanoff and colleagues treated one patient with a large GS with bowel and liver outside of the abdomen. They applied a silo with external traction for 1 month [34]. Morabito and colleagues described a traction-compression method by pulling the umbilical cord in 11 patients with GOC. Complete fascial closure was achieved in 7 out of 11 patients after 5 days of traction [33]. In a report by Mehrabi et al., fascial traction was applied via a support frame arching over the child in 6 patients with GOC and 2 patients with GS. In combination with intraabdominal tissue expanders complete closure was achieved in 14–32 days [35]. Uecker et al. reported a traction procedure without resecting the amniotic sac in GOC cases. They achieved complete closure in 7 out of 12 patients in less than 7 days (except for one patient with pulmonary hypoplasia after 40 days). In 5 patients, however, only skin closure was accomplished and a delayed fascial closure had to be done in the following months [32]. In our case series, abdominal wall closure including the fascia was accomplished in all patients. The time to fascial closure (less than 22 days in GOC and less than 11 days in GS) was within the range of the published studies stated above. However, with growing experience in the application of fasciotens^®^Pediatric, the duration of traction and time to abdominal wall closure may become shorter in the future.

Patkovsky et al. applied a traction force of 30–40% of the patients’ body weight. Other publications have not specified the degree of traction. Two authors reported, that the maximum traction force was achieved when the child’s back was gradually lifted off the bed [33, 34]. In the cases of the here presented study, traction force did not exceed 1000g. In relation to the patients’ body weight (in grams) the traction force ranged from 16- 35% in GOC and 26–46% in GS. None of the patients were lifted off the mattress. However, two tear-outs of traction sutures occurred in two GOC cases at a traction force of 32% and 34% of the patient’s body weight. The optimal traction force will need to be determined systematically in future trials.

### Amniotic sac sparing procedure

Regardless of the closing method some pediatric surgeons preserve the amniotic sac in patients with OC [29, 30, 38, 39]. In our series, the amniotic sac was initially not resected in 5 out of 10 GOC cases. As mentioned before, in 2 of these patients, a traction suture and a Gore-Tex^®^ patch tore out of the tissue and had to be replaced. Fascial closure was not affected in these patients. However, the reason for the migration of the traction sutures needs to be critically discussed. In cases in which the amniotic sac was not resected, traction sutures were placed at the rim of the defect (amnio-cutaneous line). It is important to consider, however, that the amnio-cutaneous line is not generally equivalent to the margin of the abdominal wall fascia [30, 40]. The tissue at the amnio-cutaneous line may, therefore, be less resilient for traction which may explain the tear-outs in our 2 patients.

Besides the loss of tissue resilience, the traction progress may also be less efficient when traction sutures are not placed directly to the fascia. Traction to the rim of the defect or to the umbilical cord will only transmit traction forces indirectly to the fascia. As mentioned above, Uecker et al. and Morabito et al. did not achieve fascial closure in around 40% of their cases [32, 33]. In our cohort, complete closure was achieved in all patients with amniotic sac saving procedures. However, we saw a non-significant tendency towards a longer median time to closure between patients without and with resection of the amniotic sac (9 vs. 5 days, respectively). Aljahdali and colleagues addressed the problem of securely fastening a silo to the abdominal wall without resecting the amniotic sac. They advised to dissect the fascia via a skin incision a couple millimeter proximal to the amnio-cutaneous line for a secure anchorage of the silo [40]. It will be important to pay close attention to the efficiency of different traction applications to determine the optimal procedure in the future.

### Delayed closure technique

There are many pros and cons regarding the optimal treatment strategies for large complicated abdominal wall defects. Treatment is usually guided by the pediatric surgeon`s preference and experience. Presumably, the cases presented in this study could have been successfully treated by other techniques as discussed above [19–23]. Amniotic sac epithelization (“paint and wait”) for the treatment of GOC, for example, is a preferred approach in many pediatric surgery institutions worldwide [38, 39]. It offers the advantage that no mechanical ventilation is needed in the newborn and enteral feeding can be started soon after birth [5, 39]. In addition, the non-operative approach of “paint and wait” has to be considered in children with OC-associated comorbidities such as pulmonary hypoplasia, pulmonary hypertension and severe congenital cardiac defects [38, 41–43]. “Paint and wait”, however, is a lengthy procedure lasting weeks and months before complete epithelization is achieved entailing the risks for infections and failure [30, 39]. Once epithelization is achieved, patients usually present with large ventral hernias. The reconstruction of the abdominal wall around these hernias often need meticulous plastic operations later in life with chances for failure and severe scaring [10, 24, 28, 39]. Traction-assisted fascial closure in the newborn period, as reported here, may render these drawbacks of delayed closure unnecessary. In the future, comparative studies will need to elaborate the advantages of each technique.

### General anesthesia and ventilation

In line with most reports on staged closure procedures, the majority of patients in our case series were kept under GA with paralysis during the treatment with external traction [31–35]. The median time of ventilation of 12 days was comparable to other reports on abdominal wall closure in GOC with 5–11 days of ventilation [12, 32, 33, 35, 44, 45]. In GS, the time on the respirator is reported between 2 and 7 days in staged closure and 3–5 days in primary closure [46–49]. Here, we report a median of 7 days in large complicated GS which is in the upper range of the literature cases.

In 3 of our cases, no GA was given during traction. In these patients, abdominal wall closure was achieved after 9 and 20 days in GOC and 11 days in GS. It will be of interest to investigate in a larger subset of patients whether GA with paralysis is necessary for adequate expansion of the abdominal wall or if GA and/or paralysis can be limited without compromising of the time to closure.

### Compartment syndrome

In this study, we did not experience any clinical signs of a compartment syndrome causing insufficient mechanical ventilation or anuria. In selected cases, we changed the direction of traction from vertical to diagonal after the viscero-abdominal disproportion was resolved. Diagonalization appeared to accelerate the approximation of the fascia thus facilitating tension-less closure. There was no negative influence on the intra-abdominal pressure and ventilation after diagonalization.

### Complications

There was a skin dehiscence after abdominal wall closure in GS leading to a reoperation in one of our patients. In this case, the skin had not been included in the traction appliance. At the time of fascial closure, the skin could only be closed under tension. In all other cases, the skin was included in the traction process leading to enough skin overlap during fascial closure. There was no surgical site infection and no traction-related abdominal wall necrosis, nor were there any safety-related events in the application of the traction aperture. Umbilical hernias occur at a rate of up to 18% after staged and 9% after delayed closure in GOC and is seldomly reported in GS with sutured repair [26, 47]. During the follow-up of 4 to 22 months, there was no formation of an umbilical or incisional hernia in the here reported series.

### Limitations

The small number of patients, the lack of a control group, and the short follow-up time are the main limitations of this study.

### Recommendations and further applications

The application of the traction device, fasciotens^®^Pediatric, has not been described in the treatment of GOC and GS before. Therefore, the best practice of the traction procedure using the device has to be still defined. Based on the first experiences of the 16 cases presented in this study, we summarized preliminary recommendations for the application of fasciotens^®^Pediatric in GOC and GS. However, these recommendations are not obligatory operating instructions. They should rather initiate future research to improve the treatment for GOC and GS.

Further applications of fascial traction for abdominal wall repair such as short intra-operative fascial traction (IFT; e.g. 30 min.) to achieve primary fascial closure and fascial traction for delayed abdominal wall closure of ventral hernias after “paint and wait” could also be considered in the future. In adults, successful IFT has recently been reported for large ventral hernias with a closure rate of 90% [50].

Box 1 Preliminary recommendations in the application of fasciotens^®^Pediatric in GOC and GS

- Adjust traction force to around 30% of the patient`s body weight in grams
- Expose the fascia surgically to apply the traction directly to the fascia
- In case the amniotic sac is not resected and the fascia is not exposed surgically, pay close attention to securely fasten the traction sutures to the fascia and not only the skin
- Preferably, do not apply single traction sutures directly to the fascia but interpose an alloplastic mesh between the fascia and the traction sutures to transmit the traction force evenly along the fascia lines
- Cover the abdominal wall defect with a handsewn or preformed spring-loaded silo

## Conclusion

The introduction of prosthetic silos for staged reduction have substantially advanced the treatment of congenital abdominal wall defects. Here, we report the first experience on the combination of staged closure and fascial traction with a newly designed traction device (fasciotens^®^Pediatric) for newborns with GOC and GS. The traction-assisted closure contributes to overcome the viscero-abdominal disproportion by augmenting the abdominal cavity and leads to a tension-less fascial closure by extension of the abdominal wall. The procedure requires two operations: (a) application of traction sutures with or without silo placement and (b) removal of the traction device with abdominal wall closure, making it a 2-step closure procedure. Quantification and reproducibility of the traction force by the fasciotens^®^Pediatric device may enable a standardize abdominal wall closure in large and complicated congenital abdominal wall defects in the future. Comparative studies are required to prove possible superiority over other closing methods.

## Data Availability

No datasets were generated or analysed during the current study.

## Declarations

### Image rights

The schematic image used in Fig. 1 has been modified following appropriate authorization by Fasciotens GmbH, Essen, Germany. The images in Fig. 2 belongs to the Department of Pediatric Surgery and Pediatric Urology, University Hospital Frankfurt, Germany.
